# The evolution of pace in popular movies

**DOI:** 10.1186/s41235-016-0029-0

**Published:** 2016-12-19

**Authors:** James E. Cutting

**Affiliations:** Department of Psychology, Cornell University, Uris Hall, 109 Tower Road, Ithaca, NY 14853-7601 USA

**Keywords:** Attention, Emotion, Evolution, Film style, Movies, Narrative, Pace, Popular culture

## Abstract

Movies have changed dramatically over the last 100 years. Several of these changes in popular English-language filmmaking practice are reflected in patterns of film style as distributed over the length of movies. In particular, arrangements of shot durations, motion, and luminance have altered and come to reflect aspects of the narrative form. Narrative form, on the other hand, appears to have been relatively unchanged over that time and is often characterized as having four more or less equal duration parts, sometimes called *acts* – setup, complication, development, and climax. The altered patterns in film style found here affect a movie’s pace: increasing shot durations and decreasing motion in the setup, darkening across the complication and development followed by brightening across the climax, decreasing shot durations and increasing motion during the first part of the climax followed by increasing shot durations and decreasing motion at the end of the climax. Decreasing shot durations mean more cuts; more cuts mean potentially more saccades that drive attention; more motion also captures attention; and brighter and darker images are associated with positive and negative emotions. Coupled with narrative form, all of these may serve to increase the engagement of the movie viewer.

## Significance

Experiments in cognitive psychology have shown us that rapid changes in the visual field attract our eye movements and attention. This has been demonstrated many times in the laboratory (see, for example, Theeuwes, 1991) and also when people watch movie clips (Smith, 2012). Many other laboratory studies have shown that motion also captures attention (see, for example, Franconeri & Simons, 2003), which has also been shown for people watching sections of movies (Mital, Smith, Hill, & Henderson, 2011). Still other controlled studies have shown that we have positive associations to brightness and negative associations to darkness (Valdez & Mehrabian, 1994), a finding also found for people watching short movies (Tarvainen, Westman, & Oittinen, 2015). Rapid transients (cuts), motion changes, and luminance changes have been endemic to movies for a century, and they are components of what film editors call *pace*. Nonetheless, it has taken most of that century for filmmakers to learn to fashion their tools of film style, creating a film form that couples these three physical changes (and their psychological implications) to narrative structure. A better understanding of movie structure and of movie cognition (for example, Hasson, Mallach, & Heeger, 2010; Zacks, Speer, Swallow, & Maley, 2010) continues to open a new window onto the study of mental processes as they work continuously over spans of up to 2 h and more.

## Background

[E]very shade of feeling and emotion which fills the spectator’s mind can mold the scenes in [a movie] until they appear the embodiment of our feelings.… If this is the outcome of esthetic analysis on the one side, of psychological research on the other, we need only combine the results of both into a unified principle: *the* [movie] *tells us the human story by overcoming the forms of the outer world, namely, space, time, and causality, and by adjusting the events to the forms of the inner world, namely, attention, memory, imagination, and emotion*.

Hugo Münsterberg (1916, p. 74, italics in the original)^1^

## Movies, psychology, and pace

As a psychologist, Münsterberg was overwhelmed by movies, but so were the increasingly large audiences that viewed them in the 1910s. Moreover, throughout the intervening century, movies have never lost their grip on popular culture, and, with mobile technologies, they have become more prevalent than ever. The British Film Institute (2012, p.141) estimated that the average citizen in the United Kingdom sees more than 80 films per year. Although this estimate seems overly enthusiastic, it bespeaks an impressive penetration of film media into everyday life. Popular films have become mind candy.

In addition, few art forms have changed as much as movies over the last 100 years. Some of the literature addressing this change has focused on technology (see, for example, Salt, 1992), and there are numerous textbooks and monographs that have traced cultural, economic, and political changes from the silent era to the present (see, for example, Christiansen, 1987; Kelley, 1998; Kolker, 2006; Thompson & Bordwell, 2010). There are also treatments of the changes in physical attributes of movies, both qualitative (Bordwell, 2006) and quantitative (Bordwell, Staiger, & Thompson, 1985; Cutting, DeLong, & Brunick, 2011; Cutting, DeLong, Brunick, Iricinschi, & Candan, 2011). Heretofore, however, there have been no treatments of the psychologically relevant changes in these variables as they are arrayed over the length of entire films. This article considers three dynamic patterns that are now meshed with psychological principles of attention and emotion. Before addressing them, however, let me establish some important terms from the filmmaking and film studies literatures that are pertinent to this discussion.

### Terms

*Film style* is the collection of all aspects of the craft of making movies. Filmmakers make choices about editing (varying the length and ordering shots), staging (positioning actors in front of the camera and controlling the setting behind them), framing (how much of the actors versus the background can be seen in the image, called *shot scale*), sound (controlling conversation, background noise, sound effects, and diegetic versus nondiegetic [background] music), camera motion, lighting, focus, color, and more.

*Syuzhet* is the Russian Formalist term (Bordwell, 1985; Shklovsky, 1925/1990) for the surface form of a movie – the particular lighting, sounds, and other aspects of film style that have been chosen and used over the course of a particular movie. Other terms used for this notion are *plot* (Barsam & Monahan, 2013), *discourse* (Chatman, 1980), and *narration* (Bordwell, 2008), but I find that each of these can lead to confusion – *plot* can imply synopsis and ignore film style, *discourse* can imply just the spoken language, and *narration* can imply a narrator. None of these implications is intended here. The syuzhet is the film-length, presentational, content-free form of the movie selected from among the panoply of possibilities within film style.

*Fabula* is the Russian Formalist term for the underlying story. The most common other terms used in this context are *narrative* (Bordwell, 2008) and, of course, *story* (Chatman, 1980). *Story* and *narrative* are perfectly acceptable synonyms, so I will use them here as well. The fabula is all about content. Importantly, just as the art of writing a novel is in the conversion of ideas into words on a page, Shklovsky suggested that the art of filmmaking is in the conversion of the fabula into the syuzhet (Schmid, 2010, p. 178).

*Pace* and *rhythm* are both words used in discussions of editing. They are difficult to distinguish, so I won’t try and instead will focus mostly on pace. D. W. Griffith, the esteemed early American filmmaker, may have been first to discuss pace in the context of movies. “For its ability thus to lift its patrons out of commonplace existence, and bear them hither and yon on Bagdad [sic] carpets to realms of adventure and romance, the [movie] depends upon pace” (Griffith, 1926, p. 28). Beyond this flowery prose and other than some concrete suggestions about shot duration and some oblique references to motion, Griffith was a little vague about what he meant by *pace*. Pearlman (2009, p. 47) added clarity. She suggested that *pacing* refers “to three distinct operations: the rate of cutting, the rate or concentration of movement or change in shots and sequences, and the rate of movement or events over the course of the whole film.” These tasks of editing all entail manipulations of the syuzhet.

Bordwell and Thompson (1997, p. 197; see also Polking, 1990, p. 304) drew an analogy between pace in film and tempo in music. Tempo, of course, is about time and timing, but it is also more. The musical tempo marking of *allegro* means “fast, quickly, and bright”; that of *vivace* means “lively and fast”; and these can be modified with *con fuoco* (“with fire”) and others with *misterioso* or *agitato* and dozens more. As Rao (2011, p. 17) noted, “[T]empo has three elements: rhythm, emotion, and energy.” Applied to movies, Rao’s notion would be that pace might be reflected in the temporal pattern of shot durations and in the energy reflected in a measure of motion – both part of Griffith’s (1926) and Pearlman’s (2009) analyses – but also reflected in measures relevant to emotion, the aspect of film extolled most by Tan (1996) and by Murch (2001).

Murch (2001) strongly endorsed the centrality of emotion to editing. An Academy Award-winning editor and sound designer, Murch suggested that the foremost consideration in editing is that every shot must be true to the emotional force of the narrative. This allegiance takes precedence over advancing the story, rhythmic considerations, or any concern with other attributes of film style. I focus here on one particular aspect of film style that can affect a viewer’s emotional response. It has been shown that people have positive associations to brightness and negative associations to darkness (Valdez & Mehrabian, 1994). More importantly, this has also been found for people watching short movies (Tarvainen et al., 2015). Thus, the empirical foci of this paper are those correlated with filmmakers’ notions of pace: shot duration, motion, and luminance.

## Sections of the narrative

Again, narratives are stories. All stories tend to be divisible into parts, often at different levels. This is true regardless of whether they are folktales (Mandler, 1978; Propp, 1928/1968), oral histories (Labov & Waletzky, 1967), plays (MacEwan, 1900), comics (Cohn, 2013), or movies (Field, 2005; Thompson, 1999). At one level, these parts can be called *events*, sometimes coarse-level events (Zacks et al., 2010; Zacks & Swallow, 2007). In movies, these are typically story units called *scenes* that take place in a given location, with particular characters, within a constrained amount of time (Cutting, 2014a), and that time is typically in the domain of a minute or longer. But there is also a superordinate structure to narratives.

In many cases, and particularly in movies, story form can be shown to have three or four parts, often called *acts* (Bordwell, 2006; Field, 2005; Thompson, 1999). The term *act* is borrowed from theater, but it does not imply a break in the action. Instead, it is a convenient unit whose size is between the whole film and the scene in which certain story functions occur. Because there is not much difference between the three- and four-act conceptions except that the latter has the former’s middle act broken in half (which many three-act theorists acknowledge; Field, 2005), I will focus on the four-act version.

The first act is the *setup*, and this is the portion of the story where listeners, readers, or viewers are introduced to the protagonist and other main characters, to their goals, and to the setting in which the story will take place. The second act is the *complication*, where the protagonists’ original plans and goals are derailed and need to be reworked, often with the help or hindrance of other characters. The third is the *development*, where the narrative typically broadens and may divide into different threads led by different characters. Finally, there is the *climax*, where the protagonist confronts obstacles to achieve the new goal, or the old goal by a different route. Two other small regions are optional bookend-like structures and are nested within the last and the first acts. At the end of the climax, there is often an *epilogue*, where the diegetic (movie world) order is restored and loose ends from subplots are resolved. In addition, I have suggested that at the beginning of the setup there is often a *prologue* devoted to a more superficial introduction of the setting and the protagonist but before her goals are introduced (Cutting, 2016). The prologue and epilogue roughly act symmetrically, bringing the viewer into and out of the story (see also Cohn, 2014).

Where did this four-part structure come from? Thompson (1999) suggested that it has been part of feature-length movies for a long time. In her analyses of 100 films, about half of those in the 1910s and 1920s have four acts, and from the 1930s onward in her sample, four-act movies are dominant, occurring about 90% of the time. But the existence of a four-part structure is likely much older than movies. I would claim that it has been endemic in many cultures for a long time. For example, there is a four-part narrative tradition called *kishōtenketsu* found in Japan (Berndt, 2013), and many of the Russian folktales analyzed by Propp (1928/1968) have four parts, as do other tales analyzed by Mandler (1978).

Perhaps most convincing in this domain is the work by Labov and Waletzky (1967), who showed that spontaneous life stories elicited from inner-city individuals without formal education tend to have four parts: an orientation section (where the setting and the protagonist are introduced), a complication section (where an inciting incident launches the beginning of the action), an evaluation section (which is generally focused on a result), and a resolution (where an outcome resolves the complication). The resolution is sometimes followed by a coda, much like the epilogue in Thompson’s analysis. In sum, although I wouldn’t claim that four-part narratives are universal to all story genres, they are certainly widespread and long-standing.

Thus, I assume that a general narrative form of stories was in place in our culture at the end of the 19th century, ready to be adopted by the first feature-length films of the second decade of the 20th century and then beyond. That form entails at least three, but usually four, acts of roughly equal length. Why equal length? The reason is unclear, but Bordwell (2008, p. 104) suggested this might be a carryover from the development of feature films with four reels. Early projectionists had to rewind each reel before showing the next. Perhaps filmmakers quickly learned that, to keep audiences engaged, they had to organize plot structure so that last-seen events on one reel were sufficiently engrossing to sustain interest until the next reel began.

## The evolution of film style as it corroborates narrative form

Cutting and Candan (2013) broached several issues about movies in relation to the other major arts, to culture, and to evolution. The first is that most classic art forms have been with us for a very long time. The origins of music, dance, sculpture, and painting are lost in our prehistory; theater and architecture are thousands of years old; and, although full-flowered literature had to await the printing press, it is embedded in a poetic oral tradition that is also many thousands of years old. The *longue durée* (Braudel, 2009) of the presence of these art forms suggests that they may be inseparable from what it means to be human. But their multimillennial span also makes it difficult to track their evolution because culture, languages, and economies have varied dramatically over time, and appropriate recording devices didn’t exist to register their possible development along the way.

Second, and in stark contrast, popular movies have been with us for only about 125 years. The recent development of this art form and the intense public interest it has sustained offer an intriguing possibility for studying human cognition. Although we clearly did not evolve to watch movies, it is highly likely that movies have evolved in part to fit better within our cognitive and affective preferences and capacities.

Because a reasonable number of the older movies are still with us and can be analyzed by contemporary techniques, one might be able to trace the evolution of physical structure of the art form as it has been guided by the collectivity of filmmakers’ minds, eyes, and hands.^2^ Elsewhere, Cutting, DeLong, Brunick, Iricinschi, and Candan (2011) as well as Cutting (2014b) looked at changes in movies across release years and found striking changes – shorter shot durations, more motion, and darker imagery over the last 60 years. But these whole-film measures are coarse and fail to take into account narrative structure. My intention in this article is to track those changes as they apply to the structure of movies as the narrative unfolds. To be sure, no psychological data are gathered here; only psychologically relevant physical measures on huge and lengthy stimuli that have heretofore been largely ignored are covered. In particular, I am interested in changes in syuzhets and how they have been shaped jointly by fabulas and by our cognitive and affective systems.

Previously, I demonstrated that a four-part narrative structure of the fabula has implications for syuzhet (Cutting, 2016). I explored the variations in a dozen aspects of film style as they are distributed along the length of movies. These included shot duration; shot transition types (cuts, dissolves, fades, and the like); shot scale; motion; music; luminance; character introduction; conversations; action shots; and across-shot changes in location, characters, and time. Matching the values of these variables across duration-normalized time bins in 150 movies, I then performed a principal component analysis and found that 76% of the variance could be accounted for by three orthogonal components.

Interestingly, each component aligned fairly well with one of the stimulus dimensions. For purposes of discussion, I labeled the first component as *editing* (43% of the variance). It was well captured by shot duration (eigenvector = 0.41), but also related to shot scale and character introduction. I labeled the second component as *action* (21% of the variance), and it was well represented by motion (eigenvector = 0.53) and by the location of action shots across the movies’ lengths. I labeled the third component as *lighting* (12% of the variance), well represented only by luminance (eigenvector = 0.84). Thus, there is some convergence between a three-dimensional notion of pace and the three dimensions that are most orthogonal in the analysis of film style as it saturates whole movies. The focus of this article is to address the historical changes in pace through film style measures of shot duration, motion, and emotion.

## General methods

### One hundred years of popular feature-length movies

My students and I selected 210 English-language feature-length movies released over 100 years: 10 per year at 5-year intervals from 1915 to 2015. Feature length films are defined by the Academy of Motion Picture Arts and Sciences as those at least 40 minutes in length, a duration necessary to be eligible for a regular Academy Award. These movies were among the most popular of their release years (http://www.boxofficemojo.com) or most rated on the Internet Movie Database (IMDb; http://www.imdb.com). Cutting, DeLong, and Nothelfer (2010) studied 150 of these films from 1935 to 2005 for other purposes, and Cutting, DeLong, Brunick, Iricinschi, and Candan (2011) added 10 more from 2010 with still other goals.

I next reduced these 160 feature-length movies to the 150 that were shorter than 2.5 h in duration (Cutting, 2016). This length criterion made them better candidates for having a theoretical structure proposed by Thompson (1999): four roughly equal-length sequential acts – setup, complication, development, and climax. Longer movies may have five or even six acts, often with more than one development section. For this article, the 10 longer movies were replaced with 10 others of appropriate lengths and generally equal popularity from their same release year, bringing the sample temporarily back up to 160.

For this article, in addition to the 10 replacement films, I added 50 more movies – 10 from 2015 and 10 each from 1915, 1920, 1925, and 1930 – yielding a total of 210 movies. Like the others, the newly selected movies were among the most popular of their release year on IMDb. Those from before 1930 are silent movies. I added the silent films to complete the investigation of feature-length movies in hopes of finding patterns in them that might be precursors to those of later films. I couldn’t select movies from before 1915, because the concept of feature-length film was undeveloped until the early 1910s (Pierce, 2013). Before then, almost all movies were considerably shorter than 1 h.^3^

This century’s worth of movies contains movies of many genres. Most are dramas, comedies, action films, or adventure films, with some animations, crime films, fantasies, musicals, mysteries, science fiction movies, war movies, and westerns as indicated by their first genre mention on the IMDb. Although Cutting, DeLong and Brunick (2011) and Cutting (2016) provided some analyses of genre differences, none are pursued here. All 210 films are listed in the filmography below. More importantly, I look at the changes in the temporal dynamics across release years with an eye toward revealing the evolution of movie pace and how film style has become enmeshed with narrative progression. Previously, among others, I analyzed these variables (Cutting, 2016) but was unconcerned with historical changes in pace.

### One hundred duration-normalized time bins

Because movies differ in their total duration, and in this sample they varied from 50 minutes (*Madame Butterfly*, 1915) to 147 minutes (*The Color Purple*, 1985; and *Straight Outta Compton*, 2015), each movie length was normalized.^4^ That is, each movie was assigned a unit length, the three variables measured, and the results of those analyses divided into 100 equal-duration time bins. After this, the values within like bins could be averaged across movies and a general pattern discerned.

Individual movies can show strikingly different patterns on any given measure. Thus, this type of data is quite noisy and requires pooling results over many films. For later analyses in this article, the 210 movies were divided into 4 groups – the 30 silent films (from 1915, 1920, and 1925), the 60 early sound films (from 1930, 1935, 1940, 1945, 1950, and 1955), the 60 midsample films (from 1960, 1965, 1970, 1975, 1980, and 1985), and the 60 more contemporary films (from 1990, 1995, 2000, 2005, 2010, and 2015).

*Cuts and Shot Durations*. Smith and Henderson (2008) showed that when individuals watch a movie with the task of detecting cuts, they can miss as many as 20% of them; and Over, Ianeva, Kraaij, and Smeaton (2007) reported that the best machine algorithms at that time could do only modestly better, missing about 5% of all cuts. These values are much higher than my students and I would accept. Thus, we tackled the task of finding all transitions (cuts, wipes, dissolves, and fades) by manually going through the digital versions of each movie frame by frame. The 210 movies average 108 minutes in duration, or about 155,000 frames at 24 frames/second.

**Fig. 1:**
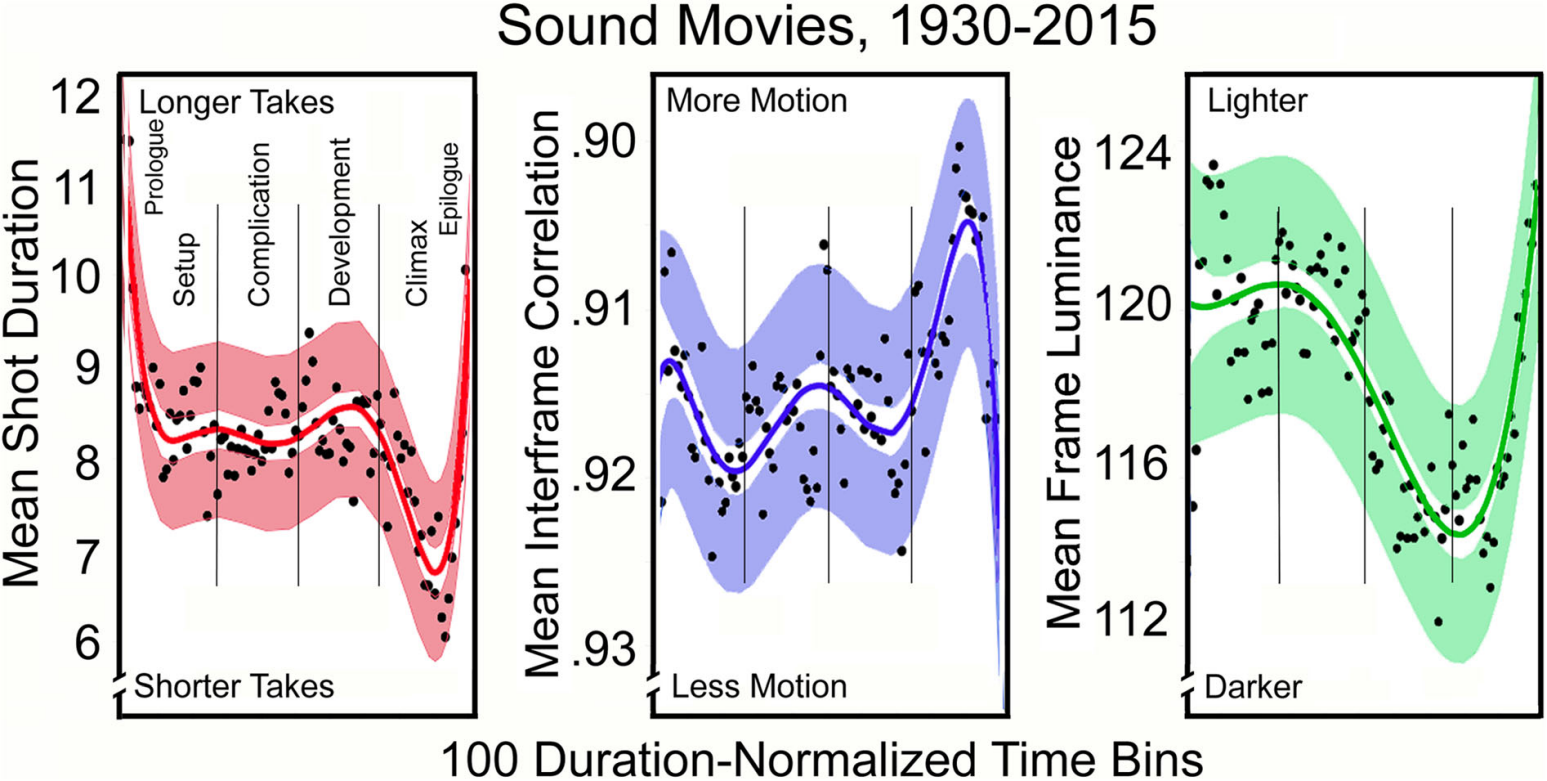
The movie-length patterns of shot duration (in seconds, *left panel*), of the extent of motion (*middle panel*), and of relative luminance (*right panel*) across 180 popular English-language movies released from 1930 to 2015. The lengths of the movies were normalized so that each measure was assessed across 100 equal-duration bins, and those values were normalized within each movie before averaging across them. Shot duration (converted back to seconds), flicker motion (converted back to mean correlations of eight-bit pixel values across frames), and luminance (converted back to the mean pixel value per frame) are three roughly orthogonal measures of film style (Cutting, 2016). Polynomial fits are shown as *darker lines* surrounded by *white areas* that correspond to the 95% confidence interval of that polynomial. *Lighter colored* regions denote the 95% confidence intervals on the data, given that polynomial. The data of each panel are also separated by *vertical lines* that suggest four narrative divisions (see Thompson, 1999): the setup (roughly bins 1–25), the complication (bins 26–50), the development (bins 51–75), and the climax (bins 76–100). Also noted are a prologue (initial and variable number of bins of the setup) and an epilogue (a variable number of bins at the end of the climax), optional units that do not appear in every film. A *take* is another name for a shot. The term *long shot* is reserved for a wide-angle shot, not a long-duration shot

Shot durations were determined in several steps. First, we found the number of the first frame of each new shot in each movie, cross-checking to assure ourselves that we found (almost) all transitions, and recorded results on a spreadsheet. The transitional frame number for dissolves and other noninstantaneous transitions (fades and wipes) was taken as its judged midpoint, thus beginning a new shot. The number of transitions that fell within each of the 100 equal-duration bins was then determined across the length of that movie. In this manner, each bin had between 0 transitions (found in at least 1 bin in 89 different movies where it was aligned with a *long take*, the term for a long-duration shot) and as many as 80 found in 1 bin in the analysis of *Avengers: Age of Ultron* (2015), a nearly 2.5-h movie with over 3200 shots. The proportion of these transitions within each bin was normalized by the number of shots in each movie (number per bin/total number of transitions), and then values in like bins were averaged across movies. These means were then rescaled back to the overall mean shot duration across all movies (7.46 seconds) as shown in Fig. 1. The average shot duration for the silent films was 7.5 seconds, that for the early sound films 10.5 seconds, that for the midsample films 7.0 seconds, and that for the more contemporary movies 4.3 seconds.

*Motion*. For a psychologist or vision scientist, motion comes in several varieties (see, for example, Borst & Euler, 2011), but these can be highly correlated in the real world and in cinema (Nitzany & Victor, 2014). Thus, I have investigated the easiest to measure – flicker motion (or flicker noise), the change in pixel values without regard to the patterns of form that change over time and space. To reduce computation time, I first downscaled each frame of all movies to a 256 × 256 array of pixels. Then each frame (if not already in black and white) was converted to 8-bit grayscale (pixel values of 0–255) by the standard MATLAB algorithm (MathWorks, Natick, MA, USA). I then adjusted the gamma of each pixel value to accord with human visual sensitivity, keeping them within the 0–255 range.

Next, I correlated the pixels in next-adjacent frames (*n* and *n* + 2) within each shot, and then I averaged the correlation values within a shot.^5^ No comparisons were made between frames across cuts. Shot averages were then assigned to the bin in which the shot began and, if that shot stretched across the next bin, to that second bin as well. The data on which analyses were performed were normalized within each movie (mean = 0.0, standard deviation = 1.0) to nullify differences across movies and emphasize only the patterns within them. Finally, as before, like-bin values were then averaged across movies.

*Luminance*. Bin luminance was determined similarly, starting with the 256 × 256-pixel, gamma-transformed grayscale values. All pixels were averaged within a frame and then averaged within a shot. Again, shot means were assigned to the appropriate bin, normalized within each movie, and averages taken across like bins.

### Analytic style: polynomial regression and cross-validation

As I have done previously (Cutting, 2016), I fit polynomial functions to the 100 bins of mean data. The sequence of these bins will be broken into 4 equal-length regions (acts), following the suggestion by Thompson (1999) that movie narratives generally consist of 4 roughly equal-length sections – the setup (here assumed to correspond to bins 1–25), the complication (bins 26–50), the development (bins 51–75), and the climax (bins 76–100). Polynomials, with their inherently curved nature, make sense in this context because there is variation in the length of the acts in a given movie. Thompson’s (1999) data suggest a mean of about 27 minutes for all acts, and a standard deviation of about 3 minutes (Cutting, 2016). Polynomial curves should accommodate these differences when averaging over the slightly different-length acts of different movies.

Nonetheless, using polynomials to fit data can lack the appropriate conservativeness in statistical analysis. This is because high-parameter count models easily lead to false-positive results. The reason for this is that all multivariable modeling is prone to overfitting, and the more parameters in the model, the more likely it will fit “too well.” Thus, model results may appear more robust than they really are because they fit with equal felicity both a theoretical pattern underlying the data and the random noise buried within it (Cutting, 2000; Pitt & Myung, 2002).

One solution to overfitting is to prefer simpler models to those that are more complex and that do not reliably produce better fits by a strict criterion. Here I will prefer a higher-order polynomial with *n* parameters only if it produces a superior fit (measured in adjusted *R* ^2^ with α < .0001) to one with *n* − 1 parameters. I will take such a result as a first confirmation of a reliable result. Unfortunately, even the use of an extremely strict criterion doesn’t really address the core problem of overfitting.

A second approach to model comparison is to penalize model complexity (models that have more parameters). There are many ways to do this (see, for example, Myung, Tang, & Pitt, 2010). For example, using adjusted *R* ^2^ values is one way to handle this problem in regression situations, a procedure I follow here. Unfortunately, this method is widely agreed to underestimate overfitting (Stauffer, 2008, p. 267). Moreover, across the plenum of multivariate analyses, there is debate about how much penalty should be levied.

A third solution, and one preferred in machine learning and elsewhere, is cross-validation (see, for example, Browne, 2000), sometimes called *within-sample prediction*. That is, one splits the data into two groups, runs the analysis on the first dataset (called the *training set*), and then uses the model form and its parameter values in a parameter-free assessment of the other set (the *test set*). If both fits are comparable and statistically reliable, this can be called a *replication*. If the fit to the test data does not yield a reliable result, then the first results are said to be due to overfitting.

In practice, it is good to do this procedure many times, so, in the cross-validation analyses performed here, I randomly split the data in half 10,000 times, then take the averages across all training and all test fits.^6^ The mean of the adjusted *R* ^2^ values across the training sets should approach the adjusted *R* ^2^ value for the means of bins in the whole dataset, and the mean of the unadjusted *R* ^2^ values across the test sets should be only a bit less. Of importance in this context is whether the test set mean *R* ^2^ value is statistically reliable. Because after a cross-validation run the training results are parameterless, the comparison between the training means and the test data means affords a two-sample correlation test on which a *t* value can be calculated. If this value is sufficiently great (again, here α = .0001), I will take such a finding as a second confirmation that the initial polynomial fit is a reliable result. In this article, I will accept results across the 100 bins *only* if the polynomial fit is confirmed in both ways,^7^ although some interesting situations arise and are worth discussing when the polynomial fits are not corroborated by cross-validation.

Finally, I will display the data in a manner that reveals both its variability and its underlying trends. To do this, I will show four things: (1) the individual mean bin data points will be in black; (2) the line of the best polynomial fit is shown in a given color (red for shot duration, blue to motion, and green for luminance); (3) the 95% confidence interval on those polynomials will surround that line as a clear region; and (4) the 95% confidence interval of the data given that polynomial can be seen in a slightly lighter color of red, blue, or green. When the polynomial fits are reliable but the cross-validation results show that these are likely due to overfitting, I will show the overall trend in the data as a solid 95% confidence interval of the data on the linear fit, even if that linear fit is not reliable. Within that region, I will show a candidate higher-order polynomial that failed cross-validation as a line in a contrasting color.

Because the focus of this article is on the dynamic patterns across the length of movies, I will try to determine when they first arose in the history of popular feature-length cinema. To do this, I will start with the 180 movies from the sound era to establish general patterns, then work backward through time in divisions within this sample, and finally consider those of the silent era to see how these patterns may have developed. Studies 1–3 will begin by exploring the global patterns across the whole of the sound-era sample; studies 4–6 will address the different eras of sound film (1995–2015, 1960–1985, and 1930–1955) for each of the dimensions; and studies 7 and 8 will consider their possible roots in silent movies (1915–1925).

## Modal pacing across the sound era, 1930–2015

Again, the three dimensions investigated here are the mean shot durations measured by transition density, the mean amount of flicker motion as measured by interframe correlations, and the mean luminance across all frames of shots in the 180 sound movies released from 1930 to 2015. Again, these are related to the film studies notion of pace, and they are the pertinent film-style variables to consider because, through componential analysis, Cutting (2016) found them to align with three orthogonal stimulus dimensions encompassing perhaps all of the various correlated dimensions of film style. Figure 1 shows three panels of mean data, each of which represents 18,000 data points culled from almost 2 trillion pixels.

### Study 1: dynamic patterns of shot durations across the length of movies

The *left panel* of Fig. 1 expands on the data from Cutting (2016, study 1; see also Cutting, Brunick, & DeLong, 2012). It presents the mean pattern of shot durations across the 100 duration-normalized time bins for the 180 movies. The *red line* shows the best-fitting sixth-degree polynomial (*t*[93] = 6.03, *p* < .0001, adjusted *R* ^2^ = .64). This fit is reliably superior to the best fits of all lower-order polynomials (adjusted *R* ^2^ = .51, .47, .33, .26, and .25, respectively, for the fifth-order polynomial down through the linear fit). Ten thousand cross-validation trials on random halves of the data yielded results for sixth-order polynomials on training data nearly comparable to those for the whole dataset (adjusted *R* ^2^ = .56), and the test results are almost as strong (unadjusted *R* ^2^ = .42, *t*[98] = 8.42, *p* < .0001, *d* = 1.7). Thus, one can be quite confident that this polynomial is a reasonable representation of the trend underlying the data.

In the *left panel*, the sequence of 100 bins is also broken into 4 equal-length regions, following the suggestion by Thompson (1999): the setup (again, roughly bins 1–25), the complication (bins 26–50), the development (bins 51–75), and the climax (bins 76–100). The two optional subsections also leave their marks on shot duration. The end of the climax often contains an epilogue where a new diegetic (film-world) order is established and loose ends are tied up (Thompson, 1999), and the beginning of the setup often contains a prologue in which the setting, the time period, and the protagonist are introduced (Cutting, 2016).

Notice two striking features within the four acts, both of which make narrative sense. First, the shot durations of the setup are initially long in the prologue but reciprocally shorten as the bin number increases (1/1, 1/2, 1/3, etc.; *t*[22] = 8.64, *p* < .0001, *d* = 3.7), helping to ease the viewer into the narrative. The subsequent shot durations are noisily varied but relatively steady through the complication and development, but then the second major feature is the decline of shot duration in the climax is followed by marked increase in the epilogue as the narrative returns to diegetic normality (quadratic trend, *t*[22] = 2.71, *p* = .02, *d* = 1.15). Cutting (2016) also reported that the shot durations in the development were slightly longer than those in the complication, but in this expanded sample, this difference, although present, is not reliable. Visual inspection suggests that the decline in shot durations starts earlier, before the end of the complication, nullifying the possible result.

As noted above, I have divided the movies into four eras – silent (1915–1925), early (1930–1955), middle (1960–1985), and near-contemporary (1990–2015). In the shot duration data across the 180 sound movies, there is a reliable bin × movie era interaction (early vs. middle vs. contemporary, *F*[2, 17,994] = 10.52, *p* < .0001), denoting that the dynamic pattern has changed over 85 years. This result presages evolutionary trends and is sufficiently important to pursue in study 4.

### Study 2: dynamic patterns of motion across the length of movies

The *middle panel* of Fig. 1, expanding on Cutting (2016, study 3), shows the general pattern of flicker motion across the length of the 180 sound movies. Again, a sixth-order polynomial, shown in *blue*, is reliably superior to others (*t*[93] = 5.17, *p* < .0001, adjusted *R* ^2^ = .57, with decreasing lower-order adjusted *R* ^2^ = .45, .34, .34, .33, and .20, respectively). Ten thousand cross-validation trials yielded similar results for the training data (mean adjusted *R* ^2^ = .47) and more than acceptable results for the test data (mean *R* ^2^ = .26, *t*[98] = 5.83, *p* < .0001, *d* = 1.18).

Again, several striking patterns are apparent that make narrative sense. In particular, there is a linear decline in motion through the setup as narratives settle down and the first conversations occur (bins 1–25, *t*[23] = 3.95, *p* = .0006, *d* = 1.64), followed by another noisy oscillation through the complication and development. This is then followed by a sharp rise in motion during the climax and a steep decline in the epilogue (quadratic fit, *t*[22] = 3.17, *p* = .005, *d* = 1.35). A bit less obvious is the reduction of the confidence interval at the beginning of the prologue. The reason for this will become clearer in study 5. Finally, as with shot durations, there was an interaction of bin × movie era (*F*[2, 17,994] = 8.16, *p* = .0003), giving another hint of the evolution of motion as a component of film style. This result is pursued in study 5.

### Study 3: dynamic patterns of luminance across the length of movies

Finally, the *right panel* of Fig. 1 expands on the results of Cutting (2016, study 4), showing the patterns of change in mean frame luminance across the length of the 180 sound movies. The 100 bins are fit well by a fourth-order polynomial (*t*[95] = 4.18, *p* < .0001, adjusted *R* ^2^ = .70), shown in *green*, and again the lower-order polynomial fits do reliably less well (adjusted *R* ^2^ = .64, .34, and .30). Cross-validation results are again quite good, with sufficiently similar results for training datasets (mean adjusted *R* ^2^ = .57) and test datasets (mean unadjusted *R* ^2^ = .26, *t*[98] = 5.93, *p* < .0001, *d* = 1.20).

There are two striking trends across this luminance profile. First, there is a strong darkening across the complication and development (bins 26–75, *t*[48] = 12.66, *p* < .0001, *d* = 3.65), and second, this is followed by a strong brightening through the climax and epilogue (bins 76–100, *t*[23] = 4.94, *p* < .0001, *d* = 2.06). Less obvious, but similar to the pattern in the *left panel* of Fig. 1, there is a marked tightening of the confidence interval during the prologue. Finally, there was a reliable interaction of bin × movie era (*F*[2, 17,994] = 5.96, *p* = .0026), suggesting for a third time that the dynamic patterns have changed over time. This effect will be pursued in study 6.

### Pace, attention, and emotion: preliminary conjectures

*Cuts and Shot Durations*. The psychological import of the overall pattern of the results of study 1 stems from the relationship between cuts and eye movements. Smith (2012, 2013) reported that cuts are generally associated with later eye movements. Viewers typically saccade back in the direction of the center of the screen. Again, this result seems consistent with filmmakers’ goals about pace. It suggests that movie viewers should be particularly attentive during the climax, driven by the content of the narrative. Similarly, the increase in cut frequency across the prologue into the remainder of the setup could serve to increase eye movements and possibly attention, bringing the viewer into the plot. Symmetrically, the decreasing pace and eye movement demands of the epilogue should help bring the viewer back out.

*Motion*. Mital and colleagues (2011) showed that motion attracts viewers’ eye movements, and hence attention, when they watch movies. This, too, suggests that engagement in the movie may be greatest during the middle of the climax. Further, and more speculatively, motion has been shown to affect the perception of the passage of time (Brown, 1995), dilating it (making it seem longer) at short durations. This might give the viewer a sense of increased processing speed, which can be associated with positive affect and well-being (Pronin, 2013). Moreover, continued fast processing speed and alternating thoughts – such as those engendered by the cross-cut scenes of a protagonist and antagonist in the climax of action films – can create anxiety (Pronin & Jacobs, 2008), which is exactly the goal of most action film narratives.

*Luminance*. Finally, consider the nadir of the luminance function. This ¾ point in movies is often called the “darkest moment” in a movie narrative (Bordwell, 2008; Keating, 2011). It occurs at the end of the development section, when events seem to be most stacked against the protagonist in trying to achieve her goals. As Cutting (2016) noted, this “darkest moment” is not simply a metaphor: On average, movies typically project their least luminous images at about this point. Similarly, the beginning of the complication, also called the *¼ point*, can be considered the brightest point (Lucey, 1996), or at least the point marked by a protagonist taking on her particular quest. Moreover, Smith (2015) noted that the protagonist’s despair at the ¾ point provides maximal contrast with the outcome of the narrative, here at the end of the epilogue, where she typically has conquered her difficulties. In luminance terms, this is literally true; epilogues are often the brightest sequence of a movie. Thus, and again, film style has appeared to mesh with the pacing goals of filmmakers. How effective this manipulation is for the viewer is unknown, but it seems to be an appropriate adjunct to the content of the narrative.

Demonstrating these three congruences – the yoking of the aspects of pace and film style to the progression of narrative events – was exactly the intent of Cutting (2016). Here, these results expand those to a broader corpus and time frame. Future research is needed to tie these patterns more directly to viewers’ responses. However, the main goal of this research effort is to trace when these congruences arose.

## Changes in pacing across the sound era, 1930–2015

With these expanded overall dynamic patterns in place, I now focus on trying to determine when they arose. Will these same higher-order polynomials be found in the patterns of all movies across the century-wide sample? The three bin × movie era interactions suggest not.

### Study 4: changes in shot duration patterns

**Fig. 2:**
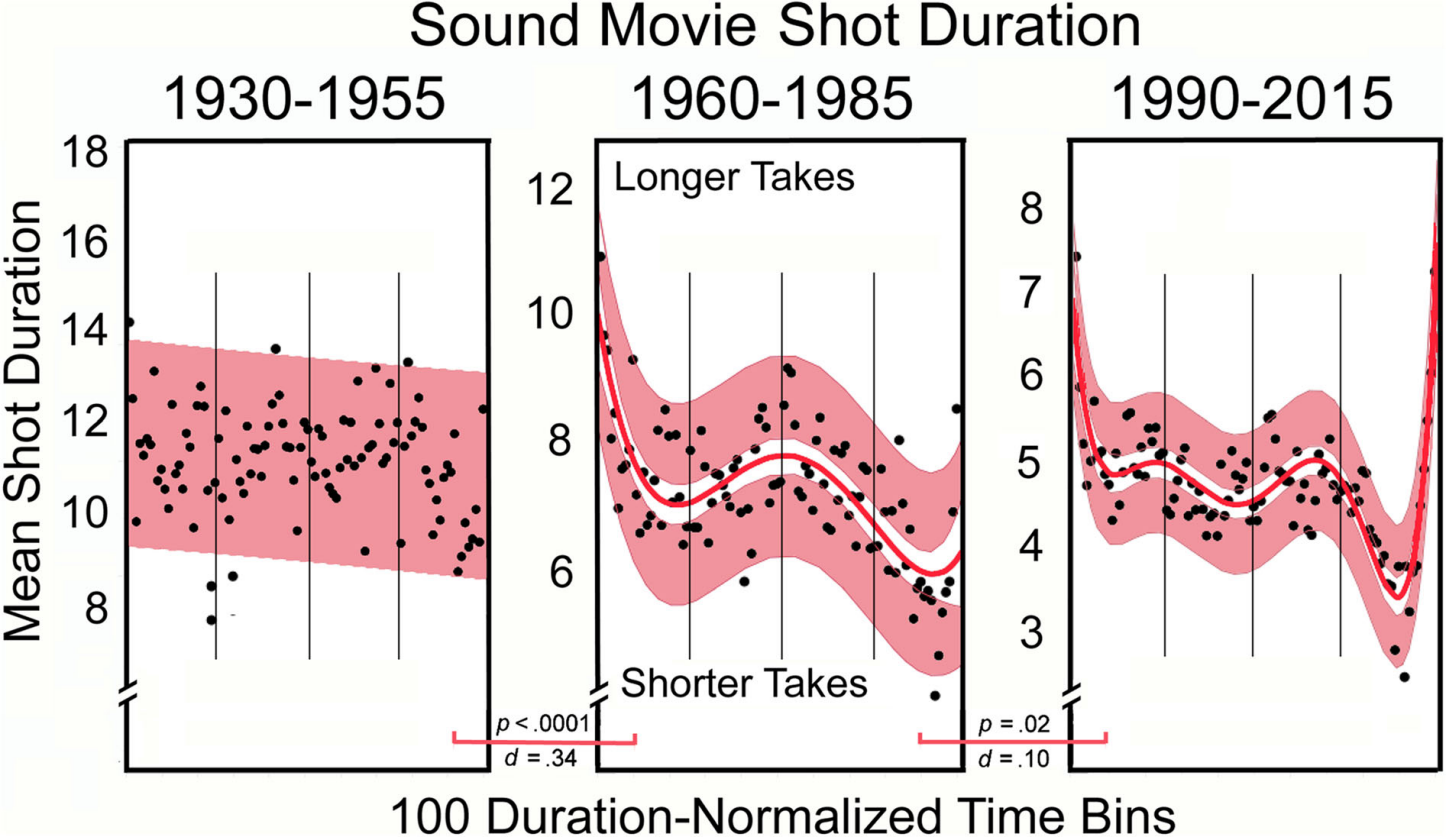
Variations in mean shot duration (in seconds) across the duration-normalized films of three different eras of popular movies. The extents of the vertical axes show equal variance in the data across the three panels. The statistical lines and regions of the *middle* and *right panels* are as they were in Fig. 1; there is no reliable pattern in the *left panel. Vertical lines* in all panels separate the bins into the four narrative sections, as in Fig. 1

*Movies from 1990 to 2015*. As shown in the *right panel* of Fig. 2, the pattern of shot durations for the 60 most contemporary movies essentially mimics that for the larger collection of all sound movies. Again, as in study 1, it is best fit by a sixth-order polynomial (*t*[93] = 8.22, *p* < .0001, adjusted *R* ^2^ = .69), which is superior to all lower-order fits (adjusted *R* ^2^ = .47, .34, .17, .17, and .16) and which cross-validation affirmed (mean training set adjusted *R* ^2^ = .58; mean test set unadjusted *R* ^2^ = .41, *t*[98] = 8.25, *p* < .0001, *d* = 1.67). The two major features of the general results in the *left panel* of Fig. 1 are here corroborated: The negative exponential decline in shot duration across the setup is again robust (*t*[22] = 7.56, *p* < .0001, *d* = 3.2), and the quadratic trend in the climax is as well (*t*[22] = 6.09, *p* < .0001, *d* = 2.6).

Thus, the pattern for the more recent movies matches quite well the average pattern of the 180 movies from 1930 to 2015. Of course, the former are contained within the latter, but, among other things, this result suggests that the overall pattern, if not pervasive across time, may at least have bin regions that are not too discrepant from those shown in the *left panel* of Fig. 1.

*Movies from 1960 to 1985*. The *middle panel* of Fig. 2 shows the pattern of shot durations from the middle chronological group of 60 sound films. This pattern is best fit by a fourth-order polynomial (*t*[95] = 4.81, *p* < .0001, adjusted *R* ^2^ = .51), which is superior to lower-order fits (adjusted *R* ^2^ = .39, .29, and .28). Cross-validation corroborated the main result (mean training set adjusted *R* ^2^ = .41; mean test set *R* ^2^ = .29, *t*[98] = 6.32, *p* < .0001, *d* = 1.28). Finally, there was a modest interaction between the bin values of the more contemporary movies (1990–2015) and those of these midrange movies (1960–1985; *F*[1, 11,996] = 5.32, *p* = .02). Thus, the patterns of the *middle* and *right panels* of Fig. 2 are a bit different.

Two features in the pattern of the middle sample movies differ from that of the latter films. These are captured in the decrease in polynomials from sixth order to fourth order. In short, the pattern is less articulated. For example, the lengthening of shots in the epilogue is not nearly so marked as in the later movies, suggesting less contrast between the action of the typical climax and the calm of the typical epilogue. In addition, the decline in shot duration is extended from the complication through to the point in the climax where the epilogue weakly turns toward longer-duration shots. This suggests that stylistically there is a bit more anticipation of action in the complication of movies of this era than in those that are more contemporary. In other words, the pattern of shot durations in movies from 1960 to 1985 is generally similar, but not identical, to those from 1990 to 2015. The overall later form is nascent but not fully present in the midsample movies.

*Movies from 1930 to 1955*. The *left panel* of Fig. 2 shows a result not seen before. There is no reliable pattern among the shot durations of the earliest sound movies. For display purposes, the data are graphed showing the 95% confidence interval on the data on a linear regression line, but even that line isn’t significant. Moreover, there was a sizable interaction of bins × movie era, early (1930–1955) vs. midsample (1960–1985; *F*[1, 11,995] = 18.5, *p* < .0001). Thus, the stylistic patterns of shot durations seen in the *left panel* of Fig. 1 appear to have developed slowly throughout the 20th century.

### Study 5: changes in motion patterns

**Fig. 3:**
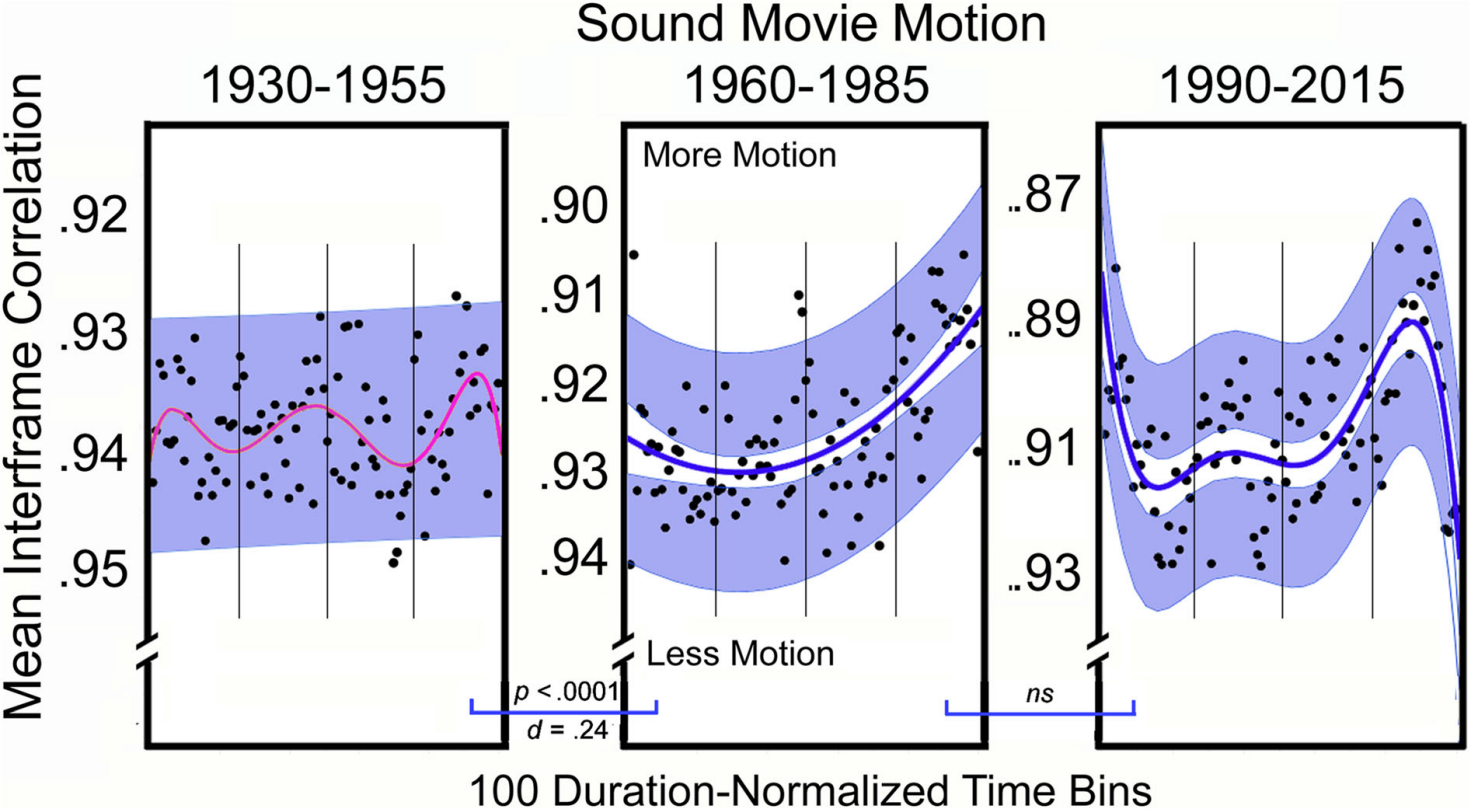
The mean flicker motion variation across the duration-normalized movies in three different eras of popular movies. The extents of the vertical axes show equal variances in the three datasets. Again, the statistical lines and regions of the *middle* and *right panels* are as they were in Fig. 1; there is no robust pattern in the *left panel*, but the *purple line* shows a reliable polynomial pattern that fails cross-validation. *Vertical lines* in all panels separate the bins into four narrative sections

*Movies from 1990 to 2015*. As seen in the *right panel* of Fig. 3, the pattern of motion across the 100 bins is best fit by a fifth-order polynomial (*t*[94] = 4.99, *p* < .0001, adjusted *R* ^2^ = .45), which is superior to lower-order polynomial fits (adjusted *R* ^2^ = .30, .29, .18, and .12). Cross-validation confirmed this pattern (mean training trial adjusted *R* ^2^ = .35; mean test trial *R* ^2^ = .16, *t*[98] = 4.32, *p* < .0001, *d* = 0.87). Again, as in study 2, there is a decline in motion across the setup (*t*[23] = 5.62, *p* < .0001, *d* = 2.34) and a quadratic trend in the climax (*t*[22] = 6.12, *p* < .0001, *d* = 2.6).

The difference in results between the larger group of sound movies and the more recent subset is again one of polynomial rank – sixth order for the 180 movies and fifth order for the 60 more recent ones. A comparison of the *middle panel* of Fig. 1 and the *right panel* of Fig. 3 shows the major difference to be in the first five or so bins, the general extent of the prologue, in both means and confidence intervals. This suggests that the newer movies are much more likely to start with greater motion than older ones, likely explaining the tightening of the confidence interval in the *middle panel* of Fig. 1. The style of contemporary movies seems better geared to engross the viewer earlier in the film, bringing her into the narrative.

*Movies from 1960 to 1985*. The motion results of the middle-era movies are quite different, as shown in the *middle panel* of Fig. 3. These data are best fit by a quadratic function (second-order polynomial, *t*[97] = 4.28, *p* < .0001, adjusted *R* ^2^ = .37), which is superior to the linear fit (adjusted *R* ^2^ = .15). Cross-validation confirms this result (mean training set adjusted *R* ^2^ = .24; mean test set *R* ^2^ = .15, *t*[98] = 4.15, *p* < .0001, *d* = 0.84).

Here we have a drop in polynomial fit from fifth-order to second-order, showing less articulation in the data. However, the difference is subtler than it might first appear; there is no reliable bin × movie era interaction (1960–1985 vs. 1990–2015 movies; *F* = 1.11, not significant). Nonetheless, gone in the fit to the midsample movie data are the systematic decreases in motion in both the prologue and the epilogue. There is no systematic interaction across the two classes of film for the prologue shown in bins 1–10, but there is a small difference in the epilogue shown in bins 90–100 (*t*[1316] = 3.07, *p* < .0022, *d* = 0.17). The expansion of the confidence interval in the epilogue suggests that movies differ in whether their motion retards at this point or not. The only residual effect is the increase in motion in the development and through the climax.

*Movies from 1930 to 1955*. The pattern of motion results for the oldest sound movies is shown in the *right panel* of Fig. 3. As there was for shot durations of this movie era, there is no reliable pattern here for motion. Data are again shown within the 95% confidence band of a linear regression, which itself is not statistically reliable. However, a sixth-order polynomial did yield a marginal result (*t*[93] = 3.17, *p* = .002, adjusted *R* ^2^ = .17), although it failed cross-validation (training set adjusted *R* ^2^ = .15, test set *R* ^2^ < 0).^8^ The point here is that buried within the essentially random data is a suspicion of a pattern that bears some resemblance to the *middle panel* of Fig. 1. Nonetheless, the interaction between bins and the early (1930–1955) and midsample (1960–1985) movies is reliable (*F*[1, 11,995] = 15.36, *p* < .0001). Thus, as with shot durations, it is clear that the general pattern of motion in movies emerged relatively slowly and became more articulated across the century.

### Study 6: changes in luminance patterns

**Fig. 4:**
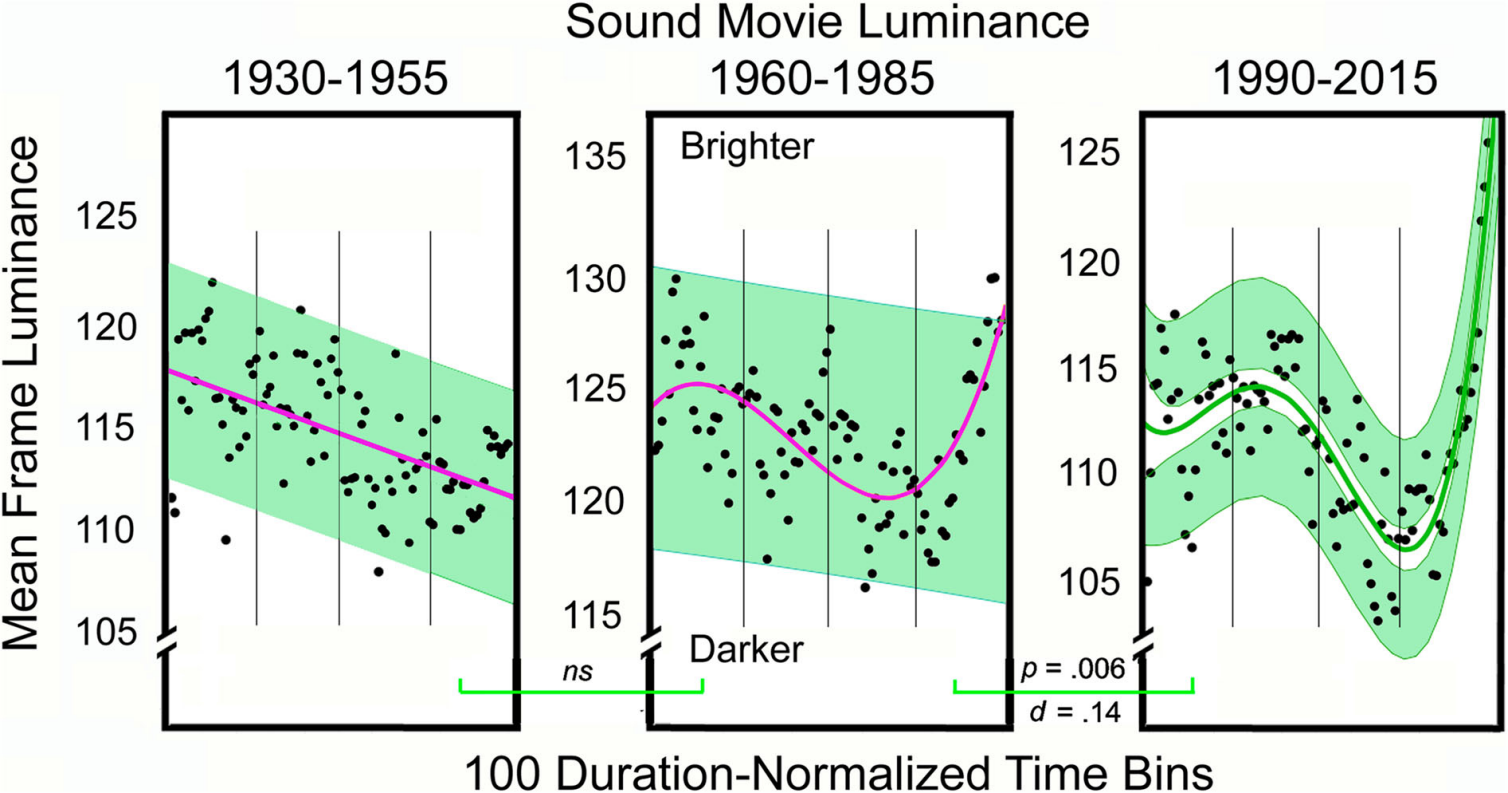
The mean luminance variation across the duration-normalized movies in three different eras of popular movies. The extents of the vertical axes show equal variance in the three datasets. Again, the statistical line and regions of the *left panel* are as they were in Fig. 1; here, there is no robust pattern in the *left* or *middle panel*, but the *purple lines* show statistically reliable patterns that failed cross-validation. *Vertical lines* in all panels separate the bins into four narrative sections

*Movies from 1990 to 2015*. The *right panel* of Fig. 4 shows the pattern of luminance across the length of movies was, as in study 3, best fit by a fifth-order polynomial (*t*[95] = 5.41, *p* < .0001, adjusted *R* ^2^ = .63), which is superior to the lower-order fits (adjusted *R* ^2^ = .52, .10, and .03). Cross-validation yielded affirming results (mean training set adjusted *R* ^2^ = .49; mean test set *R* ^2^ = .23, *t*[98] = 5.67, *p* < .0001, *d* = 1.14). Again, the decline in luminance across the complication and development is striking (*t*[48] = 8.03, *p* < .0001, *d* = 2.3), as is the linear rise through the climax (*t*[23] = 6.61, *p* < .0001, *d* = 2.75).

The pattern for the more recent movies has a polynomial fit of the same order as the entire group of sound movies. A comparison of the *right panels* of Figs. 1 and 4 shows that the reduction of the 95% confidence interval on the polynomial fit is in the prologue for both the 180 movies and the most recent 60 movies. This suggests that filmmakers’ decisions about the initial luminance composition of movies remains quite fluid. Again, the most prominent result is the progression across the complication and development of increasing darkness, matching the increasing narrative difficulty for the protagonist, followed by the striking rise in luminance as things turn out well. Again, film style corroborates narrative form.

*Movies from 1960 to 1985*. The *middle panel* of Fig. 4 shows that there is no reliable luminance pattern at all over this period. For display purposes, the data are bounded within the 95% confidence interval of a nonreliable linear fit for comparison with panels in the other figures. A third-order polynomial, the purple function within the green background and having the same decline over the complication and development, showed some promise as a successful model (*t*[96] = 4.94, *p* < .0001, adjusted *R* ^2^ = .42), but cross-validation suggested that this result was due to overfitting. The mean training set fits were reasonable (adjusted *R* ^2^ = .30), but the mean test set data yielded no support for this function (*R* ^2^ < 0). Thus, and again, there is a hint of a trend in these data similar to that found in later films. Nonetheless, the pattern of luminance across bins is different between the more contemporary movies and the midrange movies (*F*[1, 11,996] = 7.69, *p* = .006).

*Movies from 1930 to 1965*. Shown in the *left panel* of Fig. 4 are the luminance data for the movies from the earliest sound era. These show a significant linear decline (*t*[93] = 6.85, *p* < .0001, adjusted *R* ^2^ = .32), with the cross-validation results not quite corroborating that finding by my criterion (training set mean adjusted *R* ^2^ = .21; test set mean *R* ^2^ = .09, *t*[98] = 3.11, *p* < .003, *d* = 0.63). In addition, there was no reliable interaction in luminance between the early sound films (1930–1955) and those that came later in the 20th century (1960–1985; *F* < 1).

It seems possible that the hint of a linear trend over the whole film may be a precursor to the future linear trends over just the complication and the development. Nonetheless, it also appears that the full-blown pattern of luminance shown in the *right panel* of Fig. 1 did not fully emerge until the end of the 20th century, and possibly later than the shot duration and motion patterns.

## Pacing in the silent era

Perhaps nascent forms of these trends in shot duration, motion, and luminance can be found in silent movies. To be sure, direct comparison of sound movies and silent movies in this context is difficult. The reason is that about 15% of the screen time in this sample of 30 silent movies is taken up by intertitle shots – text printed in white on black. These affect the distribution of shot durations. Although the mean duration of intertitle shots in this sample (7.27 seconds) is about the same as that for live action shots (7.41 seconds), their variability is not (σ = 3.9 seconds for intertitles, 6.8 seconds for live-action shots). In other words, it is much more likely for live action shots to be very long or quite short in duration. Unsurprisingly, luminance is also affected (title slides are dark) and so is motion (ideally there is no motion in a title slide, although a bit of jitter is quite common). In addition, the distribution of the intertitles is not uniform throughout the length of the movie. Almost every silent film begins with one or more title slides, and, amassed over 20 duration-normalized bins, they become linearly less frequent as the movie progresses (*t*[18] = 4.25, *p* = .0005, *d* = 2.0).

Compositionally, there are two types of intertitles – dialogue titles and expositional titles (see Salt, 1992). The former occur within scenes and were rare before about 1913; the latter typically introduce scenes and began to appear incrementally in about 1901, often with text surrounded by artistically illustrated scroll patterns. Although dialogue intertitles are long gone from sound movies, expositional intertitles have not completely disappeared. For example, they can be found in *Goodfellas* (1990) and *Avengers: Age of Ultron* (2015) and are often present in the films of Quentin Tarantino (Cartmell, 2012).

Intertitles also had a special status in early filmmaking; scriptwriters and intertitle writers were rarely the same person, and there was even one Academy Award in 1929 given for title writing (Cartmell, 2012). In addition, intertitles were not simply add-ons after the movie was shot; they were embedded in the design of the film at its conception.

But again, for my purposes, intertitles are a nuisance. They play havoc with each of the analyses that I have undertaken and may disrupt the psychological effects that film style patterns might afford. Nonetheless, in hopes of finding some patterns that are useful to compare with the sound films, I analyzed the silent movies in two ways – first with the intertitles considered as regular shots and second with the intertitles stripped out and the remaining shots abutted as if the former did not exist.

### Study 7: silent movies with their intertitles

**Fig. 5:**
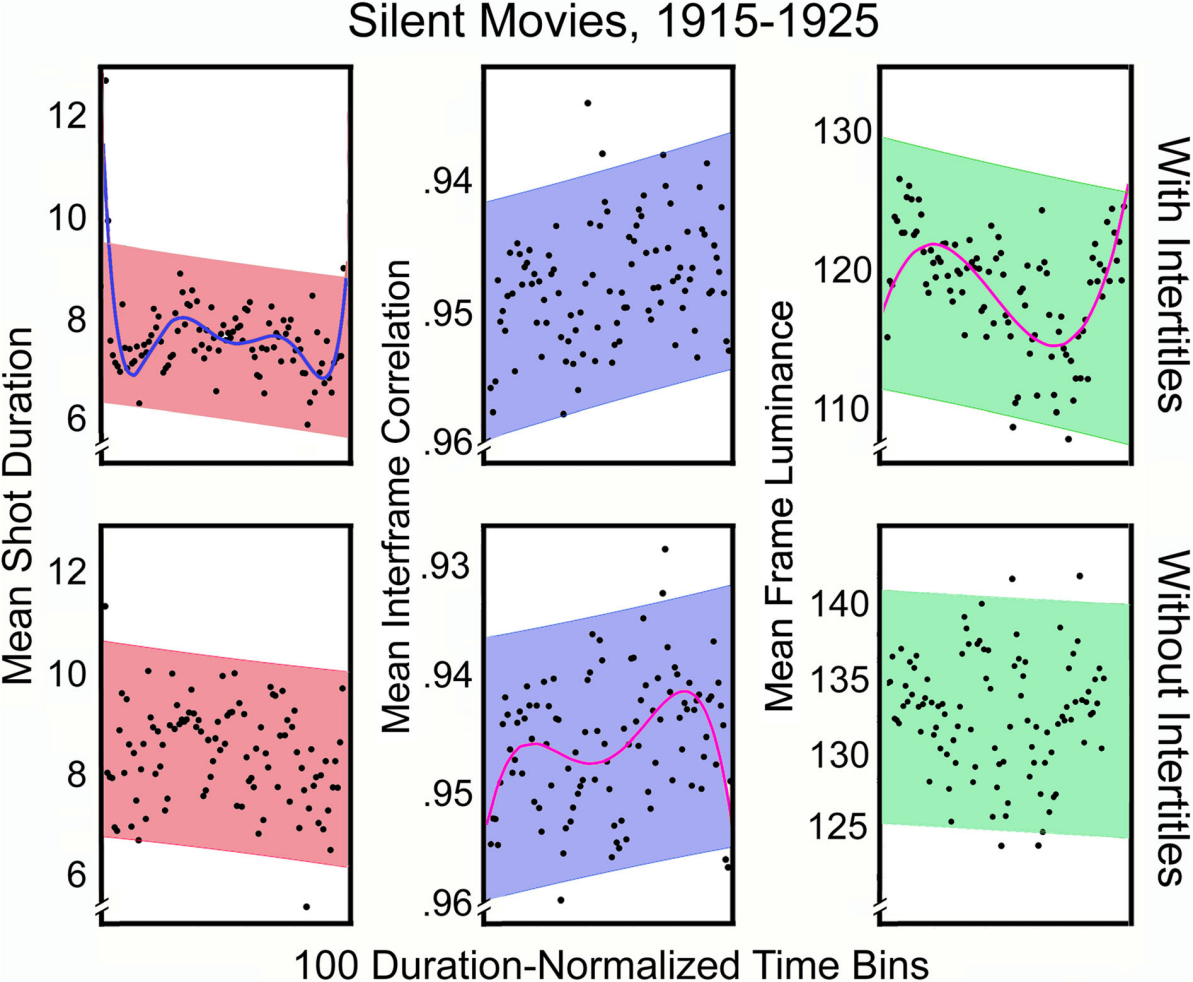
In the 30 silent movies of this sample, there are no reliable patterns of shot duration, motion, or luminance that survive cross-validation, regardless of whether the shots of intertitles are included in the analysis. However, the pattern of shot durations and luminance with intertitles and motion without intertitles showed statistically reliable polynomial fits, but these failed cross-validation

*Shot Durations*. Perhaps the biggest surprise of this set of studies is that the sixth-order polynomial was significant for the silent movies (*t*[93] = 5.51, *p* < .0001, adjusted *R* ^2^ = .43) and was superior to the lower-order polynomials (adjusted *R* ^2^ = .28, .21, .09, .07, and .03). Indeed, it only narrowly missed cross-validation by the same criterion as used elsewhere (mean training set adjusted *R* ^2^ = .28; mean test set *R* ^2^ = .07, *t*[98] = 2.71, *p* = .008). These results are shown in the *top left panel* of Fig. 5, with the *blue line* showing the fit. To be sure, the long prologue shots are often associated with complex and often multiple intertitles, and the long epilogue shots are also often similar intertitles projecting events of the narrative future. But there is even a quadratic trend in the climax and epilog (*t*[22] = 2.80, *p* = .01, *d* = 1.19).

Moreover, there isn’t even a significant interaction in the shot duration patterns between the silent movies and the contemporary sound movies of 1990–2015 (*F*[1, 8997] = 2.52, *p* > .10). Thus, there is a sense in which it took popular movies 60 years (from 1930 to 1990) to recapture the shot duration profile that silent movies seem to have had from 1915 to 1925. Nonetheless, one should be cautious inferring too much here. The silent era data are very noisy, sufficiently so that there was no reliable difference in shot duration patterns for the silent vs. the early sound movies either (*F* < 1).

*Motion and Luminance*. As shown in the *upper middle panel* of Fig. 5, there were no systematic patterns for motion. There was, however, a slight difference between motion patterns in the silent vs early sound movies (*F*[1, 8996] = 7.98, *p* = .005). This is perhaps not surprising, because in this analysis the silent movies include intertitles.

The luminance pattern in the silent era revealed a suspicion of a third-order polynomial fit in the luminance data (*t*[96] = 5.79, *p* < .0001, adjusted *R* ^2^ = .33), but although in cross-validation the training data mean is reasonable (adjusted *R* ^2^ = .15), the test data mean is not (*R* ^2^ < 0). Moreover, there was a considerable difference in the luminance pattern for silent vs early sound movies (*F*[1, 8996] = 25.7, *p* < .0001), but again the silent movie data include their intertitles.

### Study 8: silent movies stripped of intertitles

The *lower panels* of Fig. 5 show that there is no evidence of any trend across the length of silent movies in their shot durations, motion, or luminance profiles when intertitles are removed. In addition, there were also no reliable differences between the shot duration, motion, and luminance patterns across silent movies with and without their intertitles (all *F*[1, 5996] < 2.2, all *p* > .10). Somewhat surprisingly, there was a modest fourth-order polynomial fit to the motion data (*t*[95] = 2.33, *p* = .025, adjusted *R* ^2^ = .16), but this did not survive cross-validation in either the training trials (adjusted *R* ^2^ = .15) or the test trials (adjusted *R* ^2^ < 0).

One might claim that the lack of patterns in the data for silent movies with and without their intertitles is caused by mixing movies with different act structure – some with three equal-length acts and some with four. Although this is entirely possible, the beginnings of the setups and the ends of the climaxes should align in both cases, and yet there is no evidence in the data shown in Fig. 5 that would affirm this possibility.

## General discussion

### Conjectures on how pace has converged on narrative form

As a recap, let me proceed through the sequential narrative structure of movies and outline the changes in film style, when they occurred. I will also suggest possible psychological reasons for them and give examples from particular movies from this sample along the way.

*The prologue and the setup*. Some more recent movies have a “cold open” where the movie launches directly into what appears to be the middle of the narrative (*Die Hard 2*, 1990; *Erin Brockovich*, 2000; *The Social Network*, 2010). However, the typical movie of the last 70 years opens with a prologue. Shots are relatively long in duration; they are wide shots (people fill relatively little of the screen); they are occasionally coupled with dissolves rather than cuts; they may jump across time and place; they are covered with nondiegetic (background) music; they typically show few conversations; and they are often covered with title credits (Cutting, 2016). The latter might seem to account for much of this structure, but all of these features except being covered with title credits (which began in this sample in 1960) existed in the early live action shots of movies in the two decades before, when opening credits were title cards (printed lettering, typically in black on white).

In the silent era, and even into the 1930s and 1940s (*A Tale of Two Cities*, 1935; *Mutiny on the Bounty*, 1935; *Foreign Correspondent*, 1940), introductory narrative information about time and place was typically carried by one or more expository intertitles. Thus, long-duration live action shots were not used. After these intertitles, shots also had little motion; however, over the last 60 years, the prologue has gradually become one of the most active sections of movies, but still with shots relatively long in duration. These often include pans and tracking shots of the initial settings of the narrative.

The purpose of the structure of prologue shots appears to be to bring the viewer down into the narrative. Often this is literally true; in *The Hunt for Red October* (1990), for example, we first hover over a digital globe that rotates, first showing the United States and then Russia, and then in live action we look down on snowcapped mountains near Murmansk. In *MASH* (1970), we first look down on a helicopter flying over the mountains of Korea (the stand-in for Vietnam) before then looking up as it lands. In *East of Eden* (1955), we look down on the Monterey, California, coastline. Such shots create an atmosphere and inform the viewer about the locations and time periods of the narrative. Background music also helps create calm, or anxiety, or whatever emotion is appropriate to the situation (Kalinak, 2010).

There then follows an almost seamless glide into the setup and the narrative proper. Shot durations shorten but not too rapidly, and motion diminishes, but in contemporary movies again not too fast. Background music, which set the emotional tone for the beginning of the film, typically drops out, and conversations begin, often with the protagonist involved – at least 80% of all prologues introduce the protagonist (Cutting, 2016). The exogenous demands for eye movements will increase as the shot-reverse shot technique (alternately showing the characters conversing) takes over. The modal pace of the movie becomes established – the average shot duration during a conversation is typically about the average shot duration of the movies – as we learn about the goals of the characters and their circumstances. Near the end of the setup, an inciting incident often derails these goals, and a new plan of action needs to be formulated.

*Complication and Development*. Neither shot duration nor motion has any distinctive patterns in the complication and development sections. To be sure, individual movies will vary, but the normative pattern is for relative consistency across these sections, where 60% of the shots continue to show conversations, slightly more for dramas, less for action movies. Nonetheless, changes have taken place in film style that affect these narrative sections. Mean shot duration rises from the silent era (about 7 seconds) to the early sound era (11 seconds), and then shortens markedly through the midsample movies (7 seconds) to the more contemporary films (4 seconds). The decreases in shot duration in this range affect exogenous eye movements and are likely to increase attentional demands on the viewer. The mean amount of motion also increases steadily from the silent era to the present, and this, too, is likely to affect eye movements and attention, particularly in our current era of intensified continuity (Bordwell, 2006).

The major signal of dynamic change in the complication and development is in lighting, measured as overall frame luminance. Each of the four movie eras investigated here show some evidence of a decline in luminance across these sections. The evidence was weak in the silent movies, weak and nondiscriminating for the early sound movies (the decline covers the setup and climax as well), and nascent for the midsample films, but it finally crystallized in the more contemporary films. The rationale for this darkening of cinematic images appears to be connected to the travails of the protagonist as she is thwarted in her plans to achieve her goal. Indeed, the term used for the end of the development is *the darkest moment* (Bordwell, 2006; Keating, 2011; Smith, 2015). Interestingly, until recently (Cutting, 2016), there was no evidence that this was literally true. This is a case where metaphor meets preexisting reality.

*Climax and Epilogue*. The most striking evolutionary patterns in movies have occurred in the climax and epilogue. Silent movies showed some suspicion of decreased shot durations and increased movement in the climax, followed by their reversal in the epilogue, but the early sound movies showed no trace of either. The pattern reappeared, somewhat malformed, in the midsample films and became very strong in the more contemporary movies.

Why might this pattern occur? The function of the climax is to bring the protagonist to a situation where she can directly achieve her goal. In action films, this is done by physical conflict (*Spectre*, 2015); in courtroom dramas, it is typically the lead-up to a jury’s or judge’s decision (*Inherit the Wind*, 1960); in romantic comedies, it is the reconciliation of a couple (*Philadelphia Story*, 1940); in adventure films, it is working through some territorial difficulties (*Back to the Future*, 1985); and in mysteries, it can be the discovery of the solution to a crime (*Psycho*, 1960). Shorter shot durations can increase the exogenous control of eye position and eye movements; more motion attracts attention; and it seems likely that the cross-cutting of short scenes of the protagonist and antagonist in action films creates anxiety. But the increase in luminance across the climax is a signal that things should turn out well.

Of course, such a heightened state is difficult to maintain, and few movies try. The function of the epilogue is to bring the story back to diegetic normality, to tie up loose ends, and then lift the viewer (often literally with a rising crane shot, as in *Ordinary People*, 1980; *Mission*: *Impossible II*, 2000; and *Valentine’s Day*, 2010) out of the narrative. Longer duration shots and decreased motion place fewer exogenous demands on eye movements and attention, and the continued brightening of the image declares that things will remain well.

### Narrative transportation, pace, and the physical responses of viewers

Stories are the bedrock of culture. Whether by listening, reading, or viewing, we gather critically important information from stories. The phenomenon of our engagement with stories is called *narrative transportation* (Gerrig, 1993; see also Van Laer, De Ruyter, Visconti, & Wetzels, 2014). This is exactly what Griffith (1926, p. 28) had in mind when he suggested that movies “lift [viewers] out of commonplace existence, and bear them … to realms of adventure and romance.”

Busselle and Bilandzic (2009) parsed narrative engagement into four features. The first is narrative understanding, without which there can be no thorough engagement. Another is narrative presence (the feeling of having entered the story, typically at the cost of paying attention to our real-life surrounds). In this context, I’ll take narrative understanding as granted and acknowledge that narrative presence may be the most interesting component of the four.

Presence, of course, has been a key term in the domain of virtual reality since its beginning (see, for example, Barfield, Sheridan, Zeltzer, & Slater, 1995), capturing the idea that the viewer is mentally present in, and has been transported to, a different environment. Thus, it should surprise no one that when watching movies one can have changes in heart rate (Barraza, Alexander, Beavin, Terris, & Zak, 2015), changes in blood pressure (Miller et al., 2006), changes in electrodermal activity (Tsai, Levenson, & Carstensen, 2000), changes in respiratory cycling (Child et al., 2014; Tsai et al., 2000), and, when appropriate, even have oxytocin (Barraza & Zak, 2009) and endorphin release (Dunbar et al., 2016). After all, watching movies is quite a bit like watching the life around us, but with, as Alfred Hitchcock put it, the “dull bits cut out” (Truffaut, 1983, p. 103). Likely much of this physiological responding is caused by the content of the narrative, but quite likely it is augmented in the manipulation by filmmakers of the variables investigated here – patterns of shot duration, motion, and luminance.

Two of the features of narrative transportation outlined by Busselle and Bilandzic (2009) fit snugly with this view – attentional focus (lack of mind-wandering) and emotional engagement (our feeling for the characters). The changes in cut frequency and motion likely contribute to the former and luminance to the latter. Do these patterns alone affect heart rate and the other physiological measures? Empirically, this would be challenging to determine, but with enough different movies appropriately measured and enough subjects, it should be possible to answer this query.

### Film style: evolution or change?

Are these changes in patterns of shot duration, motion, and luminance across the sound era an example of an evolution or simply of change? Moreover, might the changes be simply an example of fashion, reflecting different time periods of moviemaking? Carbon (2010) noted that fashion typically follows cyclic patterns. To be sure, there is evidence of fashion in cinema. For example, the creation of dramatic long takes in some contemporary movies, particularly through the digital knitting together of separate shots, may recall a type of cinema more common to the mid-20th century. A case in point is the 6-minute-long opening “Day of the Dead” shot in *Spectre* (2015). Long takes aside, however, there is no evidence in the data presented here for a cyclic oscillation across time in measures of shot duration, motion, and luminance.

With respect to the biology, it is obvious that movies are not living entities that reproduce. Nonetheless, there is quite a lot of random variation in the movies of all eras along the dimensions investigated here. Griffith’s (1926) claims aside, I found no evidence in silent films (1915–1925) and early sound films (1930–1955) of any systematic correspondence between the structure of the fabula and the pace of the syuzhet.^9^ Yet, in the more contemporary movies (1990–2015), those correspondences are strong.

A stable narrative structure can be construed as a potential niche. In the process of natural selection, a niche serves as a “habitat” for the evolution of a species’ morphological (and other) adaptations. If the “species” is the syuzhet and the “habitat” is the general, four-part narrative structure of the fabula, I would claim that the “natural selection” across a century of filmmaking (the “random” explorations of filmmakers and their assessments of successes and failures) has brought films and their syuzhet’s morphology slowly into a species/niche-like correspondence.

Make no mistake, there may be many more such correspondences beyond the three examples investigated here, and there also may be many dimensions of change in movies for which an evolutionary perspective is unhelpful. Nonetheless, here – in the domain of whole-film pace reflected in patterns of shot durations, motion, and luminance – an evolutionary view seems unexceptionable.

### Why did these changes in pace take so long?

Whether one regards these results as a consequence of evolution or merely of a set of changes, one should ask: Why did it take 60 years and more – from 1915 or 1930 until 1990 and beyond – for the patterns shown in the *right panels* of Figs. 2, 3, and 4 to appear? In retrospect, these dynamic configurations of film style seem fairly obvious. Shouldn’t filmmakers have figured this out long ago? Continuing on the evolutionary theme, I note that cultural evolution, although sometimes quite rapid, need not be (Perrault, 2012). Three factors may have masked these changes from being obvious.

First, when editors work on movies, almost all of their efforts are on shots and scenes, not acts. Among other things, the editor’s task is to make shots carry the intended emotion, to meet the requirements of content, and to make scenes have appropriate shot durations and motion in order for pace to sensibly build up and step back (Griffith, 1926; Murch, 2001; Pearlman, 2009). Each movie has many scenes, and, if one counts cross-cut scenes separately, over the last 7 decades, there have been between 40 and several hundred separate scenes and subscenes of different durations in any given movie (Cutting et al., 2012). Editors mostly work locally, and the patterns discussed here are global to the whole film. I suspect that in a century of filmmaking practice, the local changes propagated locally, iteratively affecting nearby scenes, passing on their local constraints until a stable global pattern emerged. Such a process has been suggested in many domains of science, from vision (Saarinen, Levi, & Shen, 1997) to ecology (Pagnutti, Azzouz, & Anand, 2007).

Second, filmmaking is a craft. As a craft, its required skills are not easily penetrated in a conscious manner. The acquisition of skill is partly a trial-and-error process of exploring and developing technique, and the rules that govern the execution of the work are often more tacit than explicit (Polanyi, 1966). Collectively, editing also depends on cultural transmission, here across generations of the relatively closed society of filmmakers (Cutting & Candan, 2013, 2015). Or, more colorfully, as Walter Murch noted, “You pick up the good things that other editors are doing and you metabolize those approaches into what you are doing, and vice versa” (Ondaatje, 2002, p. 62).

Third, technology has almost certainly played a pivotal role. Cameras have gotten lighter and smaller, enhancing the use of close-ups and of motion (Bordwell, 2006). Film and now digital sensitivity have increased to make the bulky lighting equipment needed in earlier times less necessary; thus, shooting is more portable, quicker, and allows a wider range of locations. But perhaps most important are the effects of nonlinear (nondestructive) digital editing. This process, which began to be available in the late 1980s and early 1990s, can be much faster than the old cut-and-paste method for analog film, and it more easily allows for “errors” and changes. One can create and then see the results of one’s pacemaking in scenes and larger film units more readily and quickly.

### Other cinemas, other paces

Finally, the purpose of this article has been to mark evolutionary changes in the development of movie-length differences in film style as manifest over a century of popular English-language movies. Two further questions arise. The first is, would these results generalize to the national films in other languages? The answer is unclear. It is not even entirely clear that four-part fabulas would prevail in other cinemas. Thus, the analysis of a sufficiently large sample of films from Europe, Asia, or elsewhere would provide interesting and possibly different results. On the other hand, the century-long dominance of Hollywood movies in the global market suggests that there may be similar structures and effects worldwide, and thus, in pursuing any narrative-film style (fabula-syuzhet) link, it seems reasonable to have begun with English-language films.

A second question is related: Are there other stable patterns of pacing that would be plausible and effective? In other words, could the evolutionary product have been different? It should be nearly tautological to say that a well-edited movie is well-paced, but it seems probable that the form of that film’s syuzhet might systematically diverge from the general patterns found here if narratives were also quite different. *Psycho* (1960), for example, can be divided in half as if it were two movies with mostly different characters (Smith, 2009). The shower scene seems a natural (and very early) climax, and the cleanup following that scene has something like the calmness of an epilogue. Both halves of the movie have similar shot duration profiles, and each is similar to those of the *left panel* of Fig. 1 for whole movies. In addition, the second half (but not the first) has a motion pattern like that of the *middle panel* of Fig. 1, and the first half (but not the second) has a luminance pattern like that of the *right panel*. Thus, it might be possible for these results to be parts of a stable pattern if most films parsed themselves into episodic halves like *Psycho*.

## Conclusions

One response to the general results reported here is the oft-heard refrain that Hollywood movies are formulaic.^10^ Indeed they are, but this flippant critique ignores the fact that stories in all other narrative media – novels, plays, opera, comics, folktales – also generally follow very similar part-based formulae. Such formulae serve many functions, from aiding memory, to holding our attention, and offering the comfort of a familiar form (Boyd, 2009; Miller, 1990). Indeed, the familiarity and similarity of narrative forms help to bind a culture, to educate, and to entertain.

Three aspects of film style and pace that pervade the entire length of popular movies – shot duration, motion, and luminance – have developed slowly and coherently over the course of the 20th century and beyond. These now seem to better match the pacemaking intentions of filmmakers, providing information in the image that, one may speculate, also trigger increases in eye movements and other physical responses that are appropriately allied with the story line. Thus, these newer and more stable patterns should facilitate an increase in the engagement of the moviegoers.

To be sure, it would be inappropriate to say that these changes in film style make contemporary popular movies better than older ones. Good films should be judged more by the nature of their narratives than by the engagement of their viewers. The residues of engagement are likely gone shortly after the viewer has left the movie theater or turned off her DVD player, high-definition television, tablet, or smartphone. Surely, filmmakers hope that the impact of the narrative haunts the viewer’s mental life longer than that. Nevertheless, I would claim that enhancing viewer engagement through manipulations of film style that are congruent with the goals of the story makers and can serve to augment the force of the narrative; they can enhance the tendency for viewers to get lost in the story.

## Footnotes

1 The word *movie* in this quotation from Münsterberg (1916), as well as in a subsequent quote by Griffith (1926, p. 28), substitutes for *photoplay*, the common term for a feature-length movie at the beginning of the 20th century. *Photoplay* was also the name of a popular American magazine that covered movies and their stars from 1911 to 1980.

2 A serious problem considering older movies is their survival. Nitrocellulose, a highly flammable material, was the film base for movies not only in the silent era but through until 1952. Pierce (2013) estimated that only about 14% of silent feature films from 1912 to 1929 survive in their originally released form, and only 11% more come from foreign or other release sources. Survival of films between 1930 and 1952 is better, but still many are lost.

3 Five-reel movies became more or less standard in 1915, and, at 24 frames/second, a 1000-foot reel of film would last a little over 11 minutes (see Salt, 1992).

4 Total film duration was calculated from the beginning of the first live action shot until the appearance of text material at the end of movie, often “The End.” In this sample, opening credits were shown as title cards through 1955, and these were excluded from the measure. Generally, afterward opening credits were superimposed on live action shots, and these shots were included.

5 Adjacent frames were not correlated, because the publicly available DVDs for these movies often employed a digitization process that blended successive frame pairs. Sometimes this mis-sampling was endemic throughout a movie; sometimes it was only occasional.

6 A standard procedure in machine learning is to have the training set occupy 80% of the data and the test set occupy 20%. Such partitioning yields results a little different from splitting the sample in half, as I have done here. Unsurprisingly, the adjusted *R* ^2^ for the training set becomes larger as it more closely approximates the whole set, and equally unsurprisingly, the *R* ^2^ for the test set becomes smaller, given that it is less closely approximates the whole set.

7 When assessing portions of the 100-bin data, such as those bins corresponding to a given act, I use the more conventional criterion, α = .05.

8 At first blush, negative *R* ^2^ values would seem impossible, but their proper interpretation is that the particular fit is worse than a linear fit.

9 From the 1930s, pausing through the 1950s, but accelerating again thereafter, the number of shots per film has burgeoned threefold. This would mean that, given 100 equal-duration bins normalized to the length of films, there would be more noise in the number of shot durations per bin. However, given 60 movies in each era of movies used here, it seems unlikely that this could contribute to the difference in patterns.

10 As of 1 September 2016, the phrase *Hollywood movies are formulaic* had gotten 3550 hits on Google.
